# The Differential Redox Resilience of Alvelestat and Sivelestat: A Mechanistic Hypothesis for Inhibitor Performance Under Oxidative Stress

**DOI:** 10.3390/molecules31091454

**Published:** 2026-04-28

**Authors:** Maura D’Amato, Pasquale Linciano, Laurent R. Chiarelli, Giampiero Pietrocola, Paolo Iadarola, Simona Collina, Maria Antonietta Grignano, Marilena Gregorini, Teresa Rampino, Simona Viglio

**Affiliations:** 1 Department of Molecular Medicine, University of Pavia, 27100 Pavia, Italy; maura.damato01@universitadipavia.it (M.D.); giampiero.pietrocola@unipv.it (G.P.); 2 Department of Drug Sciences, University of Pavia, 27100 Pavia, Italy; pasquale.linciano@unipv.it (P.L.); simona.collina@unipv.it (S.C.); 3 Department of Biology and Biotechnologies “L. Spallanzani”, University of Pavia, 27100 Pavia, Italy; laurent.chiarelli@unipv.it (L.R.C.); paolo.iadarola@unipv.it (P.I.); 4 Research Department, IRCCS Policlinico San Matteo Foundation, 27100 Pavia, Italy; 5 Unit of Nephrology, Dialysis and Transplantation, IRCCS Policlinico San Matteo Foundation, 27100 Pavia, Italy; ma.grignano@smatteo.pv.it (M.A.G.); marilena.gregorini@unipv.it (M.G.); t.rampino@smatteo.pv.it (T.R.); 6 Department of Internal Medicine and Medical Therapeutics, University of Pavia, 27100 Pavia, Italy

**Keywords:** human neutrophil elastase (HNE), α1-antitrypsin (AAT), alvelestat, sivelestat, HNE inhibitors, chronic lung diseases

## Abstract

Human neutrophil elastase (HNE) is a key driver of inflammatory lung disorders, promoting extracellular matrix degradation and tissue damage. Although inhibitors such as Sivelestat and Alvelestat are clinically relevant, their performance within the oxidative microenvironment of diseased lungs remains poorly understood. Here, we employed an integrated in vitro and in silico approach to investigate their behavior under physiological and oxidative conditions and to provide a molecular-level interpretation. Under physiological conditions, enzymatic assays and steady-state kinetics confirmed that both compounds act as competitive inhibitors, with Sivelestat displaying higher baseline potency. Under oxidative stress, however, Sivelestat exhibited a marked reduction in inhibitory potency, whereas Alvelestat retained its efficacy. Molecular modeling and molecular dynamics simulations of native and oxidized HNE variants provided a structural rationale for this divergence. Alvelestat, as a non-covalent inhibitor, maintains stable binding despite increased flexibility of the active site, whereas Sivelestat, acting via a reversible covalent mechanism, requires a precise pre-acylation geometry. Oxidation-induced remodeling of the S1 pocket disrupts the near-attack configuration required for covalent bond formation, thereby impairing inhibition. Overall, these findings indicate that oxidative stress may selectively compromise covalent inhibition while preserving enzymatic activity, and suggest that context-dependent redox-related structural effects may represent a consideration for the design of next-generation HNE inhibitors.

## 1. Introduction

Human neutrophil elastase (HNE) is a potent serine protease stored within the azurophilic granules of neutrophils and released into the extracellular space during the inflammatory response [1,2,3]. While essential for the degradation of invading pathogens, its activity must be strictly regulated to prevent collateral damage to host tissues [4,5,6,7]. In healthy individuals, this regulation is primarily maintained by endogenous inhibitors such as alpha-1 antitrypsin (AAT) [8,9,10]. However, in severe inflammatory diseases, including chronic obstructive pulmonary disease (COPD), cystic fibrosis, acute respiratory distress syndrome (ARDS) and severe COVID-19 pneumonia, the protease–antiprotease balance collapses, leading to uncontrolled degradation of lung elastin and other extracellular matrix components [11,12,13]. A critical, yet often underestimated, driver of this pathology is the intense oxidative stress that characterizes the inflamed lung microenvironment [14,15]. Activated neutrophils generate a potent cocktail of reactive oxygen species (ROS) [15,16], including superoxide anions, hydrogen peroxide, and highly reactive oxidants such as singlet oxygen (^1^O_2_) and hypochlorous acid (HOCl) [17,18,19,20]. This “oxidative storm” exerts a dual impact: on one hand, it chemically inactivates AAT by oxidizing the critical methionine-358 residue [21,22]; on the other hand, these reactive species can directly modify HNE itself, inducing post-translational modifications and conformational changes that may alter both its catalytic efficiency and its susceptibility to synthetic inhibitors [23].

**Figure 1:**
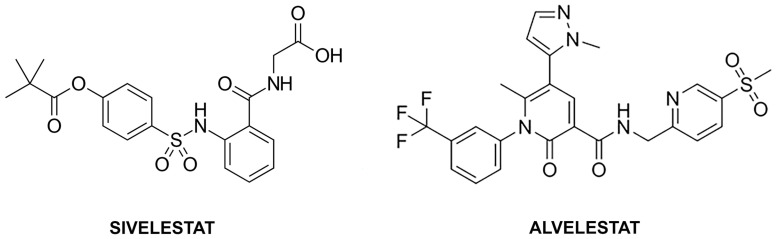
2D chemical structures of Sivelestat and Alvelestat.

Despite the clinical urgency, most drug discovery efforts targeting HNE inhibitors focus primarily on potency under optimized laboratory conditions, frequently neglecting the chemical complexity of the oxidative environment in which the enzyme operates [24,25,26]. This raises a fundamental question regarding the resilience of current therapeutics: to what extent does oxidative stress impair the binding and efficacy of different classes of inhibitors? In this context, Sivelestat, representing the class of reversible covalent inhibitors [27,28,29], and Alvelestat, a model for non-covalent inhibitors [30,31,32], provide an ideal comparative framework. The 2D chemical structures of Sivelestat and Alvelestat are reported in Figure 1.

These two compounds enable an investigation of how distinct binding strategies, one relying on precise covalent anchoring and the other on conformational adaptability, respond to structural perturbations of the enzyme target. Here, we use these systems as a case study to explore how oxidative perturbations may differentially impact inhibitor binding and to propose “redox resilience” as a context-dependent working concept rather than a generalizable property.

In this study, we address this gap by investigating the performance of Alvelestat and Sivelestat under simulated oxidative conditions. By combining in vitro enzymatic assays with molecular dynamics (MD) simulations of oxidized HNE models, we examine how oxidative perturbations in the active site differentially affect these two clinically relevant inhibitors. Our findings illustrate a potential structural rationale for the observed differences in inhibitor performance and suggest that redox resilience may represent a useful, system-dependent consideration in inhibitor design.

## 2. Results

### 2.1. Structural and Dynamic Characterization of Alvelestat and Sivelestat Binding to HNE by Molecular Modeling

To define the putative binding modes of Alvelestat and Sivelestat within the HNE catalytic site, a structure-based molecular modeling strategy combining induced-fit docking and molecular dynamics (MD) was employed. Because of the intrinsic conformational adaptability of the catalytic cleft [33,34], side-chain rearrangements upon ligand binding were explicitly considered during docking, and the most representative complexes were subsequently subjected to MD simulation to evaluate the stability and dynamics of the predicted binding modes.

**Figure 2:**
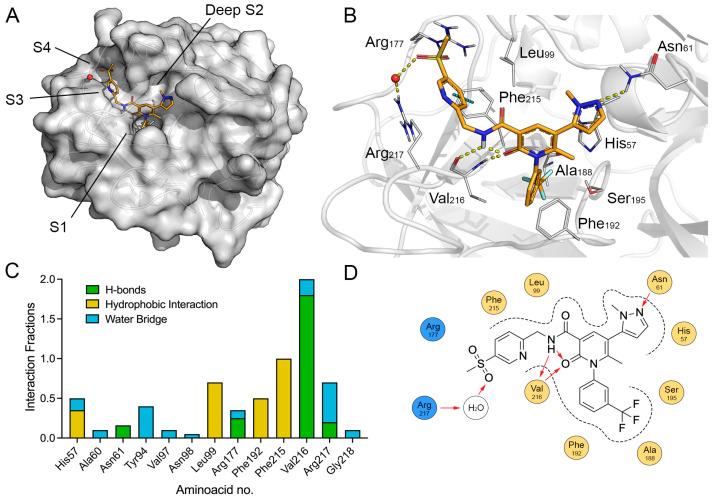
(**A**,**B**) The predicted binding mode of Alvelestat (yellow sticks) within the wt HNE binding site (PDB ID: 2Ζ7F, gray surface/cartoon). Key residues are shown as gray lines; H-bonds are indicated by yellow dashed lines. (**C**) An interaction fraction plot between Alvelestat and selected HNE residues over the MD trajectory. (**D**) A 2D interaction diagram depicting the main contacts formed by Alvelestat within the active site. H-bonds are reported as red arrows. Atoms are colored coded: oxygen in red, nitrogen in blue, sulfur in yellow.

Induced-fit docking of Alvelestat into the crystal structure of HNE (PDB ID: 2Z7F), revealed a binding mode spanning the S1–S4 subsites of the catalytic cleft. In the predicted pose, the m-(trifluoromethyl)phenyl moiety was deeply accommodated within the S1 pocket, forming hydrophobic contacts with Val216, Ala188, Phe192, and Ser195, whereas the central pyridone core anchored the ligand within the S2 sub-pocket via two hydrogen bonds with the backbone of Val216. The N-methylpyrazole moiety pointed toward a deeper S2 sub-pocket, consistent with induced local opening of this region [33,35] (Figure 2).

Cross-docking of Alvelestat into the 5ABW crystallographic structure of HNE in complex with a closely related Alvelestat analog (WQQ) produced a binding orientation closely matching that of the cognate ligand within the cleft. A structured water molecule was observed bridging the sulfonyl group of Alvelestat with Arg217, acting as a key contributor to S4 anchoring and reproducing the interaction network observed in the crystallographic complex (see Figure S1 for superimposition of the ALV predicted binding mode with the cognate ligand WQQ).

The stability of the docking-derived complex was evaluated by 100 ns MD simulation (see Figure S2 left for RMSD/RMSF analysis of the HNE_wt:ALV complexes and Video S1). During the simulation, the protein Cα backbone root mean square deviation (RMSD) fluctuated around 2.1 ± 0.8 Å, while Alvelestat RMSD remained around 1.2 ± 0.5 Å relative to the initial docking pose (Figure S2, panel A left). Ligand root mean square fluctuation (RMSF) analysis (Figure S2, panel C left) revealed limited fluctuation of the core scaffold, with RMSF value of approximately 0.5–0.6 Å for the pyridone and trifluoromethylphenyl rings. The hydrogen bonds with Val216 were maintained throughout the trajectory. A T-shaped π–π interaction with Phe215 and a water-mediated interaction involving Arg217 were also observed during the simulation, further supporting the stability of the binding mode.

**Figure 3:**
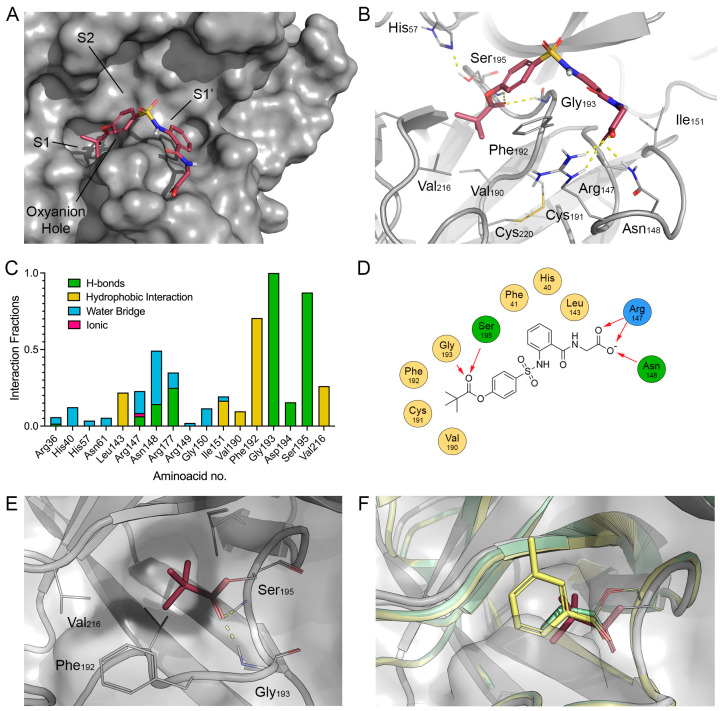
Induced-fit and covalent docking results for Sivelestat. (**A**,**B**) Most representative predicted pre-acylation complex of Sivelestat (red sticks) within the HNE binding site from MD simulation (PDB ID: 2Z7F, gray surface/cartoon with key residues shown as gray lines). (**C**) An interaction fraction plot between Sivelestat and selected HNE residues over the MD trajectory. (**D**) A 2D interaction diagram depicting the main contacts formed by Sivelestat within the active site. H-bonds are reported as red arrows. (**E**) The predicted acylated HNE complex with the pivaloyl moiety of Sivelestat from covalent docking. (**F**) Superposition of the predicted acylated complex with crystal structures of porcine pancreatic elastase acylated at Ser195 with an m-toluoylcarbonyl group (PDB ID: 8B53, yellow) and a cyclopropylcarbonyl group (PDB ID: 8B1Y, pale green). H-bonds and π−π stackings are indicated by yellow and blue dashed lines, respectively. Atoms are colored coded: oxygen in red, nitrogen in blue, sulfur in yellow.

Sivelestat is a reversible covalent inhibitor that forms an acyl–enzyme intermediate involving the catalytic Ser195. To investigate the geometry of the pre-covalent complex, induced-fit docking of Sivelestat was performed using the HNE crystal structure (PDB ID: 2Z7F). The predicted pre-covalent pose placed the pivaloyl ester group within the S1 pocket, forming hydrophobic contacts with Val216, Ala188, and Phe192. The para-substituted aromatic ring extended toward the S2 region, while the sulfonanilide moiety was oriented toward the S1′ subsite (Figure 3A–D).

Importantly, in the predicted complex, the carbonyl oxygen of the pivaloyl group formed hydrogen bonds with the backbone NH groups of Gly193 and Ser195 in the oxyanion hole. The electrophilic carbon of the ester group was positioned close to the hydroxyl oxygen of Ser195, supporting a catalytically competent pre-reactive geometry. Covalent docking was subsequently performed to model the acyl–enzyme intermediate (Figure 3E). The resulting model predicted an acylated complex in which the pivaloyl group is anchored in the S1 sub-pocket while the carbonyl oxygen is stabilized in the oxyanion hole. Superposition of the predicted complex with crystallographic structures of elastase acyl–enzyme intermediates (PDB IDs: 8B53 and 8B1Y) showed a consistent positioning of the acyl group relative to the catalytic Ser195 (Figure 3F).

The stability of the pre-covalent Sivelestat–HNE complex was evaluated by 100 ns of MD simulation (see Figure S3 for MD production analysis of the HNE_wt:SIV complex, Video S2 and Figure 3C,D). During the simulation, the Cα RMSD of the protein remained between approximately 1.1 and 1.3, indicating overall structural stability. After an initial increase corresponding to a rearrangement of the distal aryl-glycine moiety, the ligand RMSD converged to values of approximately 2.0–2.3 Å relative to the initial docking pose (see Figure S3 panels A and B for protein/ligand RMSD and ligand RMSF, respectively, for the HNE_wt:SIV complex). Throughout the entire trajectory, the Ser195Oγ-His57Nε2 is consistent with an HNE competent status (Figure S3 panel C), fluctuating around 2.7–2.8 Å [36]. Interestingly, the pivaloyl carbonyl oxygen remained engaged in hydrogen-bonding interactions with the oxyanion hole. The Bürgi–Dunitz angle defined by Ser195:Oγ–C(ester)–O(carbonyl) sampled values between 85° and 100° in approximately 28% of the simulation frames, consistent with geometries compatible with nucleophilic attack (Figure S3D). Moreover, the Gly193:N–SIV:O distance averaged approximately 2.9 Å and the Ser195:N–SIV:O distance approximately 3.1 Å, further supporting the persistence of a pre-reactive configuration (Figure S3, panels E and F).

### 2.2. In Vitro Inhibition of HNE by Alvelestat and Sivelestat

**Figure 4:**
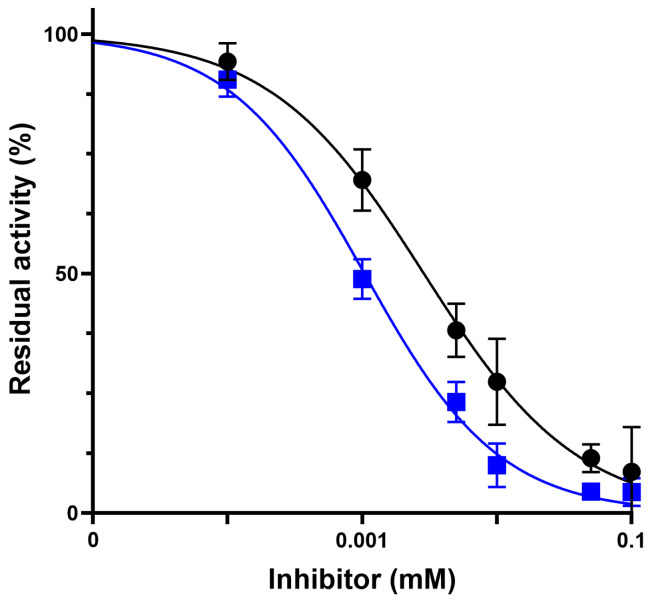
IC_50_ determination of Alvelestat (black circle) and Sivelestat (blue square) against HNE activity. Dose–response curves were obtained using an expanded set of inhibitor concentrations to improve curve fitting and definition of upper and lower plateaus. Values represent the mean ± standard deviation obtained from three separate experiments.

To validate the in silico predictions, the inhibitory effects of Alvelestat and Sivelestat on standard HNE solutions were experimentally evaluated using the synthetic peptide substrate MeOSuc-Ala-Ala-Pro-Val-pNA. Preliminary kinetic characterization of native HNE yielded a K_m_ of 0.30 ± 0.05 mM and a k_cat_ of 15 s^−1^, values that are in excellent agreement with established literature for this colorimetric assay [37], thereby confirming the reliability of the experimental setup. Subsequent inhibition assays, performed at a sub-saturating substrate concentration (0.15 mM), demonstrated that both compounds exert concentration-dependent inhibition over the tested range (Figure 4). Under these conditions, Sivelestat exhibited a higher inhibitory potency with an IC_50_ of 0.85 ± 0.08 μM, while Alvelestat showed an IC_50_ of 2.84 ± 0.33 μM.

The IC_50_ values obtained for Alvelestat are higher than those reported in some literature studies [30], which may reflect differences in assay conditions, particularly substrate concentration and experimental setup. For this reason, additional steady-state kinetic analyses were performed to derive K_i_ values under the same experimental conditions.

**Figure 5:**
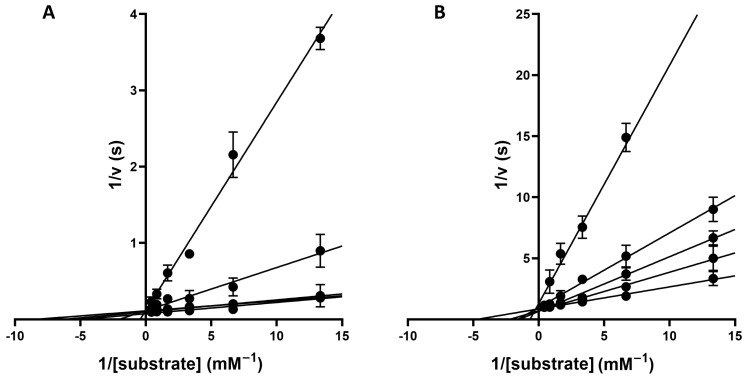
Global reciprocal plot of data from HNE steady-state kinetics analysis in the presence of different concentrations (from 0.1 μM to 100 μM) of Alvelestat (**A**) or Sivelestat (**B**). Values represent the mean ± SD obtained from three separate experiments.

To further characterize the nature of this inhibition, steady-state kinetic analyses were performed across a range of inhibitor concentrations. Global reciprocal (Lineweaver-Burk) plots (Figure 5A,B) revealed a pattern consistent with competitive inhibition for both ligands, as evidenced by the convergence of the regression lines at a common intercept on the *y*-axis. The calculated inhibition constants (K_i_) were 7.0 ± 0.9 μM for Sivelestat and 11.0 ± 0.4 μM for Alvelestat. These values were obtained by nonlinear regression of steady-state kinetic data and therefore do not rely on Cheng–Prusoff assumptions. The consistency between K_i_ and IC_50_ trends, despite the sub-saturating substrate concentration employed, supports a competitive mechanism under the experimental conditions used.

These experimental data are in agreement with the docking models, indicating that both inhibitors interact with the HNE active site, with Sivelestat showing a higher apparent potency under standard, non-oxidative conditions.

### 2.3. Impact of Oxidative Stress on HNE Inhibition and Enzyme Stability

**Figure 6:**
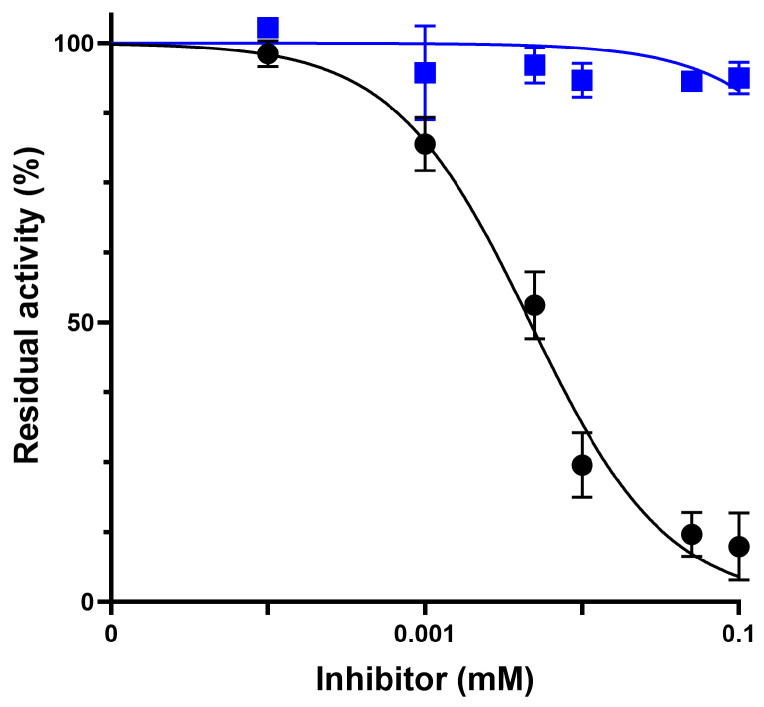
IC_50_ determination of Alvelestat (black circles) and Sivelestat (blue squares) under oxidizing conditions. Values represent the mean ± SD obtained from three separate experiments.

To evaluate the resilience of Alvelestat and Sivelestat in a biomimetic inflammatory environment, inhibition assays were conducted under oxidative stress induced by the addition of H_2_O_2_ (final concentration: 44 mM). As shown in Figure 6, Alvelestat maintained its inhibitory potency, with dose–response curves and IC_50_ values showing a modest increase compared to those obtained under native conditions (4.0 ± 0.4 μM vs. 2.8 ± 0.3 μM), remaining within the same order of magnitude. This indicates minimal impact of oxidative pre-treatment on its inhibitory activity. In contrast, Sivelestat exhibited a marked reduction in inhibitory potency following oxidative pre-treatment. While it remained a potent inhibitor of native HNE, it failed to achieve significant inhibition of oxidized HNE even at the highest tested concentration (100 μM), resulting in high residual enzymatic activity**,** thus indicating that the oxidative environment selectively disrupts its inhibitory mechanism rather than the enzyme activity per se.

To verify whether oxidative treatment affected the intrinsic catalytic competence of HNE, steady-state kinetic parameters were determined after pre-incubation with H_2_O_2_. The oxidized enzyme displayed a K_m_ of 0.32 ± 0.04 mM and a k_cat_ of 16 s^−1^, values essentially identical to those measured under native conditions. These results demonstrate that enzyme activity is preserved after oxidation, whereas the inhibitory activity of Sivelestat is strongly reduced, indicating that oxidative modifications affect the inhibition mechanism rather than enzyme catalysis itself. The values of kinetic parameters and inhibitory activity under native and oxidative conditions, which provide a direct quantitative comparison between the two inhibitors, are summarized in Table 1.

To investigate the molecular origin of the different inhibitory behaviors of Sivelestat and Alvelestat under oxidative conditions, both the intrinsic stability of the inhibitors and the structural response of HNE to oxidative modifications were examined. The intrinsic stability of both inhibitors was evaluated under oxidizing conditions. Although only Sivelestat exhibited loss of activity, both inhibitors were tested to rigorously exclude differential chemical instability as a confounding factor and to strengthen the interpretation that the observed effect originates from enzyme modification rather than compound degradation. Both compounds were incubated in the presence of 44 mM H_2_O_2_, under the same conditions used for the enzymatic assays, but in the absence of HNE, and subsequently analyzed by direct infusion ESI–MS. The full mass spectra of the samples treated with H_2_O_2_ were directly overlaid with those of the corresponding compound solutions. For both Alvelestat (Figure S4, panels A and B) and Sivelestat (Figure S4, panels C and D), the sets of *m*/*z* signals observed after oxidative treatment were identical and fully superimposable to those of the untreated controls, with no additional peaks or systematic mass shifts indicative of oxidized adducts or degradation fragments detectable within the sensitivity of the method. The absence of any new *m*/*z* peaks in the spectra strongly argues against the formation of abundant oxidized products under the applied conditions.

On the protein side, HNE contains eight cysteine residues arranged into four disulfide bridges, which are potential targets of oxidative modifications (see Figure S5 for the positions of the disulfide bridge relative to the catalytic site). Cysteine thiols and disulfide bonds can undergo stepwise oxidation to sulfenic, and sulfinic and, under severe or prolonged oxidative conditions, may irreversibly evolve toward sulfonic acid (cysteic acid) derivatives [38,39]. Among the disulfide bridges present in HNE, the Cys42-Cys58 and Cys191-Cys220 pairs were selected for explicit modeling because of their spatial proximity to the active site (Figure S5 left). Two oxidized models for HNE were therefore prepared. In the first HNE oxidized model (HNE_OX1), the Cys42-Cys58 disulfide lies in proximity to the catalytic triad, whereas in the second HNE oxidized model (HNE_OX2) the Cys191-Cys220 disulfide bridge is located near the S1 pocket. The Cys42-Cys58 in HNE_OX1 and the Cys191-Cys220 disulfide bonds in HNE_OX2 were replaced by two cysteic acid residues. These models were designed to probe how localized, terminal oxidation at structurally sensitive disulfides could differentially impact catalytic geometry and S1 pocket architecture, rather than to exhaustively describe all possible oxidation states of HNE.

Both apo HNE_wt and apo oxidized models HNE_OX1 and HNE_OX2 were subjected to 20 ns MD simulations to evaluate the structural effects of these modifications on the catalytic triad and on the S1 pocket (see Figure S6 for RMSF analysis of HNE_wt and oxidized models HNE_OX1 and HNE_OX2 and for Ser195:Oγ-His57:Nε2 distance fluctuation). In the HNE_OX1 model, oxidation of the Cys42-Cys58 bridge produced detectable changes in the geometry of the catalytic triad during the MD trajectory. The distance between Ser195:Oγ and His57:Nε2 shifted toward longer values compared with the wild-type enzyme, and the corresponding hydrogen bond was largely disrupted relative to the native state (Figure S6, panels B and C) [40]. In addition, Ser195:Oγ became engaged in a persistent hydrogen bond with Cys42-SO_3_H, promoting a reorientation of the serine away from His57 throughout the simulation and indicating a perturbation of the catalytic machinery. In the HNE_OX2 model, oxidation of the Cys191-Cys220 disulfide bridge did not produce major alterations in the catalytic triad geometry during the MD trajectory (Figure S6, panel D). The Ser195:Oγ-His57:Nε2 distance remained within the hydrogen-bonding range for most of the simulation and showed fluctuations comparable to those observed in the wild-type enzyme. However, the RMSF profile of the protein Cα atoms indicated increased mobility in the region surrounding the Cys191-Cys220 bridge, which is located at the periphery of the S1 subsite (Figure S6, panel A), suggesting localized rather than global structural perturbations. In addition, a pronounced RMSF peak is observed for residues 142–148 (Figure S6, panel A), corresponding to a highly mobile loop. This loop lies in the vicinity of the binding site but does not form part of the S1 pocket or the oxyanion-hole; its elevated RMSF, observed also in the HNE_wt, therefore reflects intrinsic flexibility rather than oxidation-induced remodeling of catalytically relevant residues.

The observation that HNE_OX2 model preserves the core geometry and catalytic competence of the triad, while inducing localized softening of the S1 region, is consistent with our kinetic data showing that oxidized HNE retains near native K_m_ and k_cat_ values (Table 1). Comparable profiles and geometric trends were obtained for the three independent replicas of each complex, indicating that the observed effects of oxidation on local flexibility and pre-reactive geometry are reproducible within the explored timescale.

To evaluate the effect of this structural modification on inhibitor binding, Sivelestat was docked into the HNE_OX2 model, and the resulting complex was subjected to a 100 ns MD simulation and compared to MD production for HNE_wt:SIV complex (see Figure S7 for RMSD/RMSF analysis of the HNE_OX2:SIV complex and comparison with HNE_wt:SIL complex and Video S3). During the first 10 ns of the trajectory, the ligand maintained a binding orientation similar to that observed in the wild-type enzyme. As the simulation progressed, the pivaloyl ester group gradually moved away from its deeply buried position within the S1 pocket, as reflected by the increased ligand RMSF, especially for the pivaloyl moiety (Figure S7 center and right, panel C). This displacement was accompanied by disruption of the hydrogen-bond interactions between the ester carbonyl oxygen and the oxyanion hole NH groups of Gly193 and Ser195. During the trajectory, the ligand did not re-establish a stable geometry comparable to the initial pre-covalent complex, indicating a loss of the pre-reactive configuration required for covalent inhibition. Concomitantly, the S1 subsite exhibited increased local flexibility, with enhanced motion of the loop region containing the oxidized Cys191–Cys220 pair (Figure S7 right, panel B), further supporting a mechanism in which localized structural perturbations impair the formation of a productive enzyme–inhibitor complex. Conversely, no significative variation in the binding mode for Alvelestat was observed during MD production for the HNE_OX2:ALV complex (see Figure S2 center for RMSD/RMSF analysis of the HNE_OX2:ALV complex and Video S4).

### 2.4. Cytotoxicity Profile in Human Alveolar Epithelial Cells

**Figure 7:**
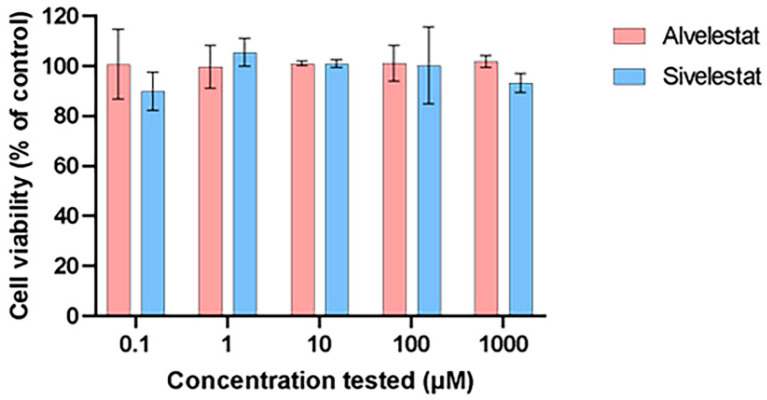
Cytotoxicity evaluation of Alvelestat and Sivelestat in A549 cells. Cell viability was assessed after incubation with increasing concentrations of compounds (up to 1000 µM) using the resazurin assay. Resorufin fluorescence was measured at λ_exc_ = 520 nm and λ_em_ = 580 nm. Data are expressed as a percentage of the untreated control (100%) and represent the mean of three independent experiments.

To evaluate the safety profile of Alvelestat and Sivelestat in a lung-relevant model, cytotoxicity assays were performed using the human alveolar basal epithelial cell line A549. Metabolic activity was quantified via the resazurin reduction assay across a broad concentration range (0.1 to 1000 µM). As shown in Figure 7, cell viability remained consistently close to 100% for both compounds throughout the tested range. These results indicate that neither Alvelestat nor Sivelestat induces detectable cytotoxic effects in A549 cells, even at concentrations far exceeding their IC_50_ values.

## 3. Discussion

Inhibition of human neutrophil elastase is a cornerstone in the management of inflammatory lung diseases, such as COPD, ARDS, cystic fibrosis and severe COVID-19 pneumonia. In these conditions, dysregulated HNE activity drives the uncontrolled degradation of the extracellular matrix, making the development of selective inhibitors a vital therapeutic strategy. Among these, Sivelestat and Alvelestat represent two of the most clinically relevant compounds. This study clarifies their binding modes and inhibitory mechanisms through an integrated in silico and in vitro approach, providing a mechanistic framework to explain their performance under both physiological and pathological conditions.

Docking and MD analysis indicate that Alvelestat engages HNE through a non-covalent, induced-fit binding mode stabilized by a distributed interaction network across the S1–S4 subsites, resulting in a stable complex. The ligand is deeply accommodated within the S1 pocket while maintaining additional interactions toward solvent-exposed regions, including water-mediated contacts, supporting stable anchoring without requiring strict geometric constraints. In contrast, Sivelestat behaves as a reversible covalent inhibitor and relies on a more stringently organized pre-acylation geometry. Docking and MD simulations show that its binding mode positions the reactive carbonyl within the oxyanion hole, where hydrogen-bond interactions with backbone NH groups stabilize a near-attack configuration compatible with nucleophilic attack. This pre-reactive geometry is consistently maintained throughout the trajectory, supporting efficient covalent engagement.

The enzymatic assays are consistent with these structural observations. The kinetic parameters obtained for native HNE are in close agreement with previously reported values [36], supporting the robustness of the experimental setup. Both inhibitors displayed potent, concentration-dependent inhibition, with Sivelestat exhibiting higher baseline potency than Alvelestat, consistent with their distinct inhibitory mechanisms. The higher apparent efficiency of Sivelestat likely reflects the formation of a covalent acyl–enzyme intermediate [25,29,41], whereas Alvelestat relies on an extensive network of non-covalent interactions to stabilize the complex. Steady-state kinetic analysis confirmed that both compounds behave as competitive inhibitors, with inhibition constants consistent with the observed IC_50_ trends. However, their performance diverged markedly under oxidative conditions. While Alvelestat maintained its inhibitory capacity with only minor variations, Sivelestat exhibited a pronounced reduction in potency. Importantly, steady-state kinetic parameters of oxidized HNE remained essentially unchanged, demonstrating that the enzyme’s intrinsic catalytic competence is preserved under oxidative conditions. To exclude direct oxidation of the inhibitors, their intrinsic chemical stability was evaluated under the same oxidative conditions used in the enzymatic assays. The lack of detectable degradation or oxidation products for either compound following incubation with H_2_O_2_ revealed by ESI-MS analysis indicates that the observed effects are unlikely to arise from direct modification of the small molecules. These results therefore point to oxidation-induced structural alterations of HNE as the most plausible origin of the altered inhibitory response. Although the present data do not resolve individual kinetic steps, the combined enzymatic and computational results indicate that oxidative perturbations primarily affect the formation of a productive pre-acylation complex rather than preventing ligand binding. Accordingly, the preservation of catalytic activity alongside the loss of inhibition is thus most plausibly explained by disruption of the geometric requirements for covalent engagement.

A central point concerns the physiological relevance of the oxidative conditions employed. Although the H_2_O_2_ concentration exceeds the transient micromolar levels measured in extracellular fluids, ROS generated during the neutrophil oxidative burst reach high local fluxes and include more reactive oxidants such as HOCl and ^1^O_2_ [17,18,19,20], capable of inducing irreversible modifications including cysteine overoxidation to sulfonic acid [39,42,43]. Accordingly, the use of 44 mM H_2_O_2_ should be interpreted as an accelerated model of cumulative oxidative damage rather than a direct representation of in vivo conditions. Within this framework, Sivelestat appears more sensitive to oxidative perturbations due to its requirement for a precisely organized pre-acylation geometry, whereas Alvelestat retains inhibitory activity, consistent with a non-covalent, induced-fit mechanism that better tolerates structural rearrangements of the catalytic pocket.

To elucidate the molecular basis of these differences, we investigated the impact of disulfide overoxidation on HNE’s structure and inhibitor binding. Comparison of the native enzyme with HNE_OX1 and HNE_OX2 variants, in which Cys42–Cys58 and Cys191–Cys220 were converted to sulfonic acid residues, revealed distinct stability profiles. MD simulations of HNE_OX1 showed perturbations of the catalytic machinery, including disruption of the Ser195–His57 interaction and reorientation of Ser195, consistent with partial decoupling of the catalytic pair and suboptimal nucleophile activation. Given that HNE retains measurable catalytic activity under oxidative conditions, these findings suggest that extensive Cys42–Cys58 overoxidation is unlikely to represent the predominant in vivo modification.

By contrast, HNE_OX2 maintained the core architecture of the catalytic triad, remaining comparable to the wild-type enzyme and consistent with preservation of basal catalytic activity. The loop bearing Cys191–Cys220 displayed increased mobility and the S1 pocket exhibited modest widening, highlighting this bridge as a plausible redox-sensitive hotspot that may modulate the S1/oxyanion-hole geometry without fully compromising catalysis. Modeling of Sivelestat binding to HNE_OX2 revealed a progressive destabilization of the Michaelis-like pre-acylation geometry during MD simulations. Although the ligand initially retained a wild-type-like binding mode, the pivaloyl ester gradually shifted from its S1 position, with concomitant loss of key hydrogen bond interactions and failure to re-establish a stable near-attack geometry. Notably, these changes occurred without disruption of the catalytic triad, supporting a selective impairment of the pre-reactive geometry rather than global enzymatic dysfunction. These observations suggest a mechanism in which localized remodeling of the S1/oxyanion hole region may interfere with the geometric requirements for covalent engagement by Sivelestat, while preserving catalytic competence. It is important to emphasize that the HNE_OX1 and HNE_OX2 structures should be regarded as hypothesis-driven models of disulfide overoxidation. The exact pattern and extent of cysteine oxidation remain to be fully elucidated. Within these limitations, our models provide a mechanistically consistent framework linking localized, terminal oxidation at Cys42–Cys58 or Cys191–Cys220 to the experimentally observed preservation of catalytic activity and selective impairment of covalent inhibition.

On the other hand, the resilience of Alvelestat likely lies in its non-covalent, induced-fit binding mechanism. Unlike covalent agents, Alvelestat exhibits conformational adaptability that allows for the reorganization of its interaction network even under perturbations of the active site. By engaging a broader and more flexible binding surface rather than relying on a single covalent anchor point, Alvelestat could compensate for local oxidative damage in one sub-pocket by strengthening interactions in adjacent regions. This structural robustness provides a plausible rationale that while oxidative stress disrupts the rigid geometry necessary for acylation, the inherent flexibility of the induced-fit model may ensure sustained efficacy in the aggressive microenvironments of diseased lungs.

It is worth noting that the classical docking and molecular dynamics are well suited to describe geometrical pre-organization, local flexibility and non-covalent interaction patterns, but only provide an indirect picture of covalent inhibition. In particular, the covalent docking model of the Sivelestat acyl–enzyme complex yields a plausible structural arrangement of the adduct, and is not intended to describe the full kinetics or thermodynamics of bond formation. Likewise, the oxidative modifications introduced in the HNE_OX1 and HNE_OX2 models mainly modulate the local electrostatic and steric environment without explicitly representing redox-coupled changes in charge distribution or redox potentials. Accordingly, our conclusions focus on the relative stability of pre-reactive complexes and the propensity to maintain near-attack geometries in native versus oxidized HNE, rather than on a complete quantum mechanical description of covalent chemistry or redox equilibria. More advanced approaches, such as QM/MM calculations or atoms-in-molecules analyses, would be required to probe bond making/breaking and charge polarization in oxidized HNE in a more rigorous manner and are envisaged as valuable directions for future work.

Another central question is whether the irreversible conversion of HNE cysteine residues into sulfonic acid groups represents a physiologically plausible event. Terminal oxidation of protein thiols is well documented in chronic inflammation; for instance, the trioxidation of serum albumin at the conserved Cys34 residue is a recognized biomarker of cumulative oxidative stress in metabolic and systemic disorders [44,45]. In the lung environment, where COPD and ARDS patients exhibit a persistent “oxidative burst,” HNE is exposed to high concentrations of reactive oxidants, which drive rapid and hierarchical thiol oxidation pathways [15]. While cysteine–sulfenic acid formation is typically transient and reversible [46], sustained ROS exposure can promote further oxidation to stable sulfonic acid derivatives, leading to irreversible structural and biophysical alterations of the protein [39,47]. These local perturbations are likely critical for Sivelestat, which requires precise spatial alignment of their electrophilic warhead within the catalytic machinery. In this context, substrate turnover can accommodate a certain degree of conformational flexibility, whereas covalent reversible inhibition depends on stricter geometric constraints to enable efficient acyl-enzyme formation. Accordingly, even subtle oxidative distortions of the S1 pocket may impair the pre-reactive alignment required for covalent engagement. In contrast, Alvelestat maintains its inhibitory efficacy under the same conditions, likely due to its ability to tolerate the increased flexibility of the binding site, indicating that this binding mode may better accommodate oxidative perturbations in this system.

Collectively, these findings suggest that oxidative stress may exert a dual impact on HNE: while the global structural integrity of the enzyme appears largely preserved, localized distortions within the catalytic pocket may selectively impair the molecular machinery required for reversible covalent inhibition.

## 4. Materials and Methods

### 4.1. Reagents

Standard HNE was purchased from Abcam (Cambridge, UK). The synthetic non-peptide inhibitors Alvelestat and Sivelestat were obtained from Selleckchem (Munich, Germany) and Abcam (ab146184), respectively. The standard p-nitroaniline (p-NA) and the peptide substrate MeOSuc-Ala-Ala-Pro-Val-pNA used for the determination of HNE activity were obtained from Bachem GmbH (Weil am Rhein, Germany). Unless otherwise stated, all other analytical grade reagents were purchased from Sigma Aldrich (St. Louis, MO, USA). All buffers used were prepared using double-distilled water obtained with a Millipore (Bedford, MA, USA) Milli-Q purification system.

### 4.2. Molecular Modeling

Molecular docking was performed using Flare (Cresset, version 5). The X-ray crystal structure of HNE in complex with the SLPI (secretory leukocyte protease inhibitor) peptide (PDB ID: 2Z7F; resolution: 1.70 Å) and the reversible inhibitor WQQ (PDB ID: 5ABW; resolution: 1.85 Å) was retrieved from the RCSB Protein Data Bank. Chain A was retained for all calculations, as it provided complete and high-quality structural information. Missing atoms and side chains (i.e., for the 142–148 loop) were rebuilt using Flare’s preparation tools. Protonation states of ionizable residues were assigned at pH 7.4, and hydrogen atoms were added. The protein was subjected to restrained energy minimization using the MMFF94 force field to relieve local steric clashes. The chemical structures of Alvelestat and Sivelestat were generated using the molecule editor of Flare and protonation and tautomeric states at physiological pH (7.4) were computed using Ligand Prep tool. The binding site was defined by centering the docking grid on the hydroxyl oxygen atom of the catalytic Ser195 residue and extended to include the S1–4 and S1′–2′ subsites (for docking against the structure of HNE in complex with SLPI, PDB ID: 2Z7F) and the cavity occupied by the co-crystallized ligand (for docking against the structure of HNE in complex with WQQ, PDB ID: 5ABW). Default parameters were used for ensemble docking. To assess the reliability of the docking workflow, the co-crystallized ligand WQQ was extracted from the 5ABW structure and re-docked into the prepared HNE binding site. The resulting pose reproduced the experimental binding conformation with a root-mean-square deviation (RMSD) of 0.95 Å, thereby validating the docking setup for subsequent ligand placement and scoring. For each ligand, ten poses were generated. Docked poses were visually inspected within Flare to examine key molecular interactions such as hydrogen bonding, electrostatic complementarity, and hydrophobic contacts.

### 4.3. Molecular Dynamics Simulations

Molecular dynamics (MD) simulations were performed using GROMACS 2025.2 release on an NVIDIA GeForce RTX 4060 GPU. The protein–ligand complex was solvated in a cubic box using the TIP3P water model, with a minimum distance of 1.0 nm between the complex and box edges. The system was neutralized by adding Na^+^ counterions. Energy minimization was performed using the steepest descent algorithm until the maximum force was less than 1000 kJ/mol/nm. The system was equilibrated in two phases. First, NVT (constant Number of particles, Volume, and Temperature) ensemble equilibration was conducted using Brownian dynamics at 10 K for 100 ps, followed by 12 ps with position restraints on solute heavy atoms. Second, NPT (constant Number of particles, Pressure, and Temperature) ensemble equilibration was performed without heavy atom restraints at 10 K for 12 ps and at 300 K for 24 ps. Pressure was maintained at 1 bar using the Parrinello–Rahman barostat and temperature coupling was achieved using the modified Berendsen thermostat. Production MD simulations were conducted with a 2 fs time step under NPT conditions (1 bar, 300 K). 1000 frames per simulation were saved for analysis. Root mean square deviation (RMSD) and root mean square fluctuation (RMSF) analyses were performed using GROMACS analysis tools to assess the stability of the protein-ligand complex and identify regions of conformational flexibility.

To investigate the structural effects of oxidative stress on HNE, the initial structure was derived from the crystallographic model of HNE in complex with SILP (PDB ID: 2Z7F). To model cumulative oxidative damage on disulfide bridges, the Cys42–Cys58 (HNE_OX1) and Cys191–Cys220 (HNE_OX2) disulfide bonds were manually cleaved and each cysteine residue was converted to cysteic acid (–SO_3_H), corresponding to the terminal sulfonic oxidation state of cysteine. The protein was solvated in a cubic TIP3P water box and neutralized with appropriate counterions. The system was minimized as reported above, and production MD was carried out at 310 K in the NPT ensemble with periodic boundary conditions. For each protein–ligand complex three independent production trajectories were generated starting from the same equilibrated structure but using different randomized initial seeds. The trends reported were consistently observed across all replicas. Analyses were performed using GROMACS analysis tools and Python 3.12.

### 4.4. HNE Enzyme Activity, Steady-State Kinetics and Inhibition Assays

HNE activity was assessed through a colorimetric assay based on the hydrolysis of the synthetic peptide substrate MeOSuc-Ala-Ala-Pro-Val-pNA [37]. To determine the steady-state kinetic parameters, 0.4 μg of HNE (corresponding to a final concentration of approximately 0.05 μM) were added to an incubation buffer consisting of 50 mM Tris-HCl, pH 7.8 containing 500 mM NaCl. The reaction was initiated by adding a fixed volume (20 µL) of intermediate substrate working solutions to the enzyme–buffer mixture, reaching a final reaction volume of 200 µL. These working solutions were prepared by diluting a 20 mM substrate stock solution in DMSO with incubation buffer, ensuring that the final DMSO concentration remained constant at 1% (*v*/*v*) across all tested substrate concentrations (ranging from 0.075 to 2.4 mM). This approach was applied to all samples, including blanks, to maintain a uniform solvent environment. The mixtures were incubated at 37 °C for 45 min. Preliminary time-course experiments confirmed that product formation remained linear over the incubation period under the experimental conditions used. The reaction was then quenched with 20 μL of 0.45 M trichloroacetic acid (TCA). After cooling on ice for 30 min, the samples were centrifuged at 11,500 rpm for 10 min to remove precipitated proteins. The concentration of p-nitroaniline (p-NA) released into the supernatant was determined by measuring the absorbance at 410 nm. All assays were performed in triplicate, and the kinetic parameters were determined by fitting the experimental data to the Michaelis–Menten equation using GraphPad Prism 8.0 software (GraphPad Software, San Diego, CA, USA).

In parallel, HNE activity was investigated in the presence of Alvelestat and Sivelestat. Initially, IC_50_ values were determined by assaying the enzyme activity under steady-state conditions using a fixed substrate concentration of 0.15 mM and varying inhibitor concentrations ranging from 0.1 μM to 100 μM. Stock solutions of both inhibitors were prepared in DMSO and diluted with incubation buffer to obtain intermediate working solutions. A fixed volume of these working solutions was added to each reaction mixture so that the final DMSO concentration remained constant at 1% (*v*/*v*) across all inhibitor concentrations. IC_50_ values were estimated according to Equation (1), where A_[I]_ and A_[0]_ represent the enzymatic activity in the presence and absence of the inhibitor, respectively.

(1) $$A_{\left[I\right]}=A_{\left[0\right]}\times \left(1-\frac{\left[I\right]}{\left[I\right]+{IC}_{50}}\right)$$

Subsequently, the inhibition constants (K_i_) were determined independently from IC_50_ values by performing steady-state kinetic assays at varying concentrations of both substrate and inhibitors. Data were analyzed using an adapted equation for competitive inhibition [48] (Equation (2)) implemented in GraphPad Prism 8 software.

(2) $$v=\frac{V_{max}[S]}{\left[S\right]+K_{m}\left(1+\frac{\left[I\right]}{K_{i}}\right)}$$

### 4.5. Inhibition Assays in the Presence of H_2_O_2_

HNE activity under oxidative conditions was determined using the colorimetric assay described above. Briefly, HNE was pre-incubated with 1 μL of 30% (*w*/*v*) H_2_O_2_ (final concentration 44 mM in a total reaction volume of 200 μL) for 60 min at 37 °C. The oxidative conditions used in this study were designed to promote cumulative oxidative modifications of HNE rather than to reproduce physiological ROS concentrations, allowing simulation of oxidative stress in chronically inflamed tissues where repeated ROS exposure leads to progressive protein oxidation. The kinetic parameters of oxidized HNE were determined as described above. Subsequently, inhibition assays were performed by initiating the reaction with substrate (final concentration 0.15 mM) in the presence of increasing concentrations of inhibitor (0.1–100 μM). The reaction mixtures were then incubated at 37 °C for 45 min and then terminated by the addition of 20 μL of 0.45 M TCA. Samples were cooled on ice, centrifuged, and analyzed as described in Section 2.4.

Residual enzymatic activity (%) was calculated relative to HNE activity in the absence of inhibitors, set as 100%. The lowest observed activity under the experimental conditions was taken as the maximal inhibition achieved within this assay range.

### 4.6. Mass Spectrometric Assessment of Inhibitor Stability Under Oxidative Conditions

The chemical stability of Alvelestat and Sivelestat under oxidative conditions was evaluated by direct-infusion electrospray ionization mass spectrometry (ESI–MS). Mass spectrometry analysis was performed using an Advion Interchim Scientific expression^®^ Compact Mass Spectrometer (CMS) (Advion Interchim Scientific, Ithaca, NY, USA) equipped with an ESI source. Stock solutions of each inhibitor were prepared in DMSO and diluted in an assay buffer to a final concentration of 100 μM (DMSO ≤ 1% *v*/*v*). Aliquots were incubated in the presence or absence of H_2_O_2_ (44 mM final concentration) for 30 min at 37 °C, matching the oxidative treatment used in the enzymatic assays. After incubation, samples were diluted 1:1 (*v*/*v*) with water containing 0.1% formic acid and directly analyzed in both positive- and negative-ion mode. Mass spectra were analyzed with Mass Express software version 5.1.

### 4.7. Determination of Silvelestat and Alvelestat Cytotoxicity on A549 Cell Monolayers

The cytotoxicity of Sivelestat and Alvelestat was evaluated using the human alveolar epithelial cell line A549. Cells were obtained from the American Type Culture Collection (ATCC, Manassas, VA, USA) and cultured according to the supplier’s instructions. Cells were maintained in DMEM supplemented with 10% fetal bovine serum (FBS) and seeded into sterile 96-well plates at a density of 1 × 10^5^ cells per well, allowing for the formation of a confluent monolayer prior to treatment. After 24 h incubation at 37 °C (5% CO_2_), the culture medium was replaced with 200 μL of fresh medium containing ten-fold dilutions of the test compounds, ranging from 0.1 to 1000 µM.

After a 16 h exposure period, cell viability was assessed via the resazurin reduction assay [49]. Briefly, 30 μL of a 0.01% resazurin solution (Sigma-Aldrich, St. Louis, MO, USA) was added to each well and incubated for 4 h at 37 °C. The reduction of resazurin to resorufin by metabolically active cells was quantified using a ClarioSTAR microplate reader (BMG Labtech, Ortenberg, Germany), with excitation and emission wavelengths set at 520 nm and 580 nm, respectively. All experiments were performed in triplicate, and cell viability was expressed as a percentage relative to untreated control wells.

## 5. Conclusions

This study provides a structural and mechanistic comparison of the inhibition of human neutrophil elastase by Sivelestat and Alvelestat under both physiological and oxidative conditions. While Sivelestat exhibits higher baseline potency under physiological conditions, its covalent inhibitory mechanism appears more sensitive to oxidative stress, likely due to the disruption of the precise geometric requirements for acyl–enzyme formation. The experimental data therefore indicate that oxidative stress reduces the efficacy of the covalent inhibitor without markedly impairing the intrinsic catalytic competence of the enzyme. The computational analyses suggest that localized oxidative modifications near the active site may increase the flexibility of the S1 subsite and perturb the geometry of the oxyanion hole region, thereby potentially interfering with the spatial requirements for acyl–enzyme formation. In contrast, Alvelestat retains inhibitory efficacy following oxidative pre-treatment of HNE, consistent with its non-covalent binding mode, which appears less sensitive to moderate structural perturbations of the catalytic pocket.

Together, these results suggest that redox-dependent structural perturbations of the target enzyme may influence inhibitor performance and should therefore be considered when evaluating inhibitor efficacy under oxidizing conditions. In this system, the data point to a functional decoupling between preserved enzymatic activity and impaired covalent inhibition under oxidative stress. From a broader perspective, this case study suggests that conformationally adaptable inhibitors may better tolerate oxidative perturbations, although this observation is currently limited to the systems investigated. More generally, these findings highlight the importance of evaluating inhibitor performance not only under ideal biochemical conditions but also under environments that more closely reflect pathophysiological oxidative stress. Future studies on additional inhibitors and more complex biological models will be necessary to assess the generality and translational relevance of these findings.

## Figures and Tables

**Table 1:** Values of kinetic parameters and inhibitory activity under native and oxidative conditions.

	K_m_ (mM)	k_cat_ (s^−1^)	IC_50_ Sivelestat (μM)	IC_50_ Alvelestat (μM)	Residual Activity at Maximum Inhibitor Concentration (%)
Native HNE	0.30 ± 0.05	15	0.85 ± 0.08	2.84 ± 0.33	<20% (both inhibitors)
HNE under oxidizing conditions	0.32 ± 0.04	16	>100 μM	4.0 ± 0.41	>80% (Sivelestat) <20% (Alvelestat)

## Data Availability

The original contributions presented in this study are included in the article/Supplementary Material. Further inquiries can be directed to the corresponding author.

## Supplementary Materials

The following supporting information can be downloaded at: https://www.mdpi.com/article/10.3390/molecules31091454/s1, Figure S1: Superimposition of the Alvelestat predicted binding mode by docking calculations with the cognate ligand WQQ co-crystalized with HNE (PDB ID 5ABW): Figure S2: Molecular dynamics analysis of the Alvelestat (ALV) complex with HNE in the wild-type (wt) and oxidized (HNE_OX2) forms; Figure S3: 100 ns MD simulation of the wtHNE–Sivelestat pre-complex; Figure S4: Mass spectra of Alvelestat and Sivelestat under control and oxidative conditions; Figure S5: Surface representation of HNE highlighting the positions of the four disulfide bridges (yellow) relative to the catalytic site; Figure S6: Effect of disulfide bond overoxidation on HNE flexibility and catalytic geometry; Figure S7: Molecular dynamics analysis of the sivelestat (SIL)–HNE complex in wild-type (wt) and oxidized (HNE_OX2) forms. Video S1: 100 ns MD simulation for HNE_wt in complex with Alvelestat; Video S2: 100 ns MD simulation for HNE_wt in complex with Sivelestat; Video S3: 100 ns MD simulation for HNE_OX2 model in complex with Sivelestat; Video S4: 100 ns MD simulation for HNE_OX2 model in complex with Alvelestat.

## Abbreviations

The following abbreviations are used in this manuscript:

AAT	α1-antitrypsin deficiency
ACN	Acetonitrile
ARDS	Acute respiratory distress syndrome
CID	Collision-induced dissociation
COPD	Chronic obstructive pulmonary disease
DA	Diode array
DMEM	Dulbecco’s modified eagle medium
DMSO	Dimethyl sulfoxide
ESI	Electrospray ionization
FBS	Fetal bovine serum
HNE	Human neutrophil elastase
IC_50_	Half maximal inhibitory concentration
K_i_	Inhibitory constant
LTQ	Linear trap quadrupole
LC-MS/MS	Liquid chromatography tandem mass spectrometry
MD	Molecular dynamics
ROS	Reactive oxygen species
RMSD	Root mean square deviation
RMSF	Root mean square fluctuation
RP-HPLC	Reverse-phase high performance liquid chromatography
SLPI	Secretory leukocyte protease inhibitor
TCA	Trichloroacetic acid
TIP3P	Transferable Intermolecular Potential 3-Point
