# Supplementary material for: The Differential Redox Resilience of Alvelestat and Sivelestat: A Mechanistic Hypothesis for Inhibitor Performance Under Oxidative Stress
Source: Molecules. 2026 Apr 28;31(9):1454. doi: 10.3390/molecules31091454 (PMC13165411; doi:10.3390/molecules31091454)
Supplement: Supplementary file 1 [file molecules-31-01454-s001.zip › Supplementary Materials.pdf]

Supplementary Materials

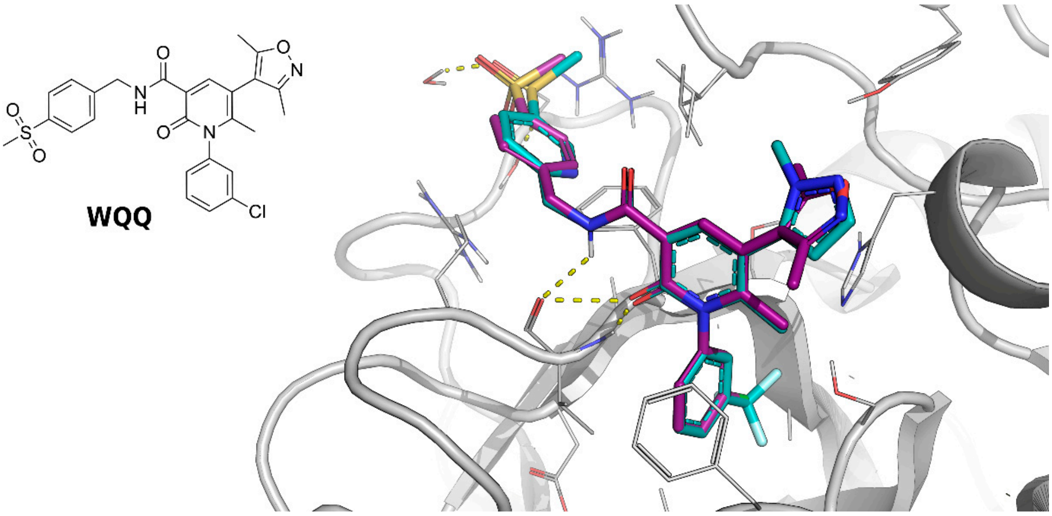

Figure S1. Superimposition of the Alvelestat predicted binding mode by docking calculations with the cognate ligand WQQ co-crystalized with HNE (PDB ID 5ABW). Alvelestat and WQQ are represented in teal and purple sticks carbon, respectively. HNE is displayed in gray cartoon and main interactive amino acids in lines. Atoms are color-coded with oxygen in red, nitrogen in blue, sulfur in yellow, chlorine in green and fluorine in cerulean. H-bonds are represented in dotted yellow lines.

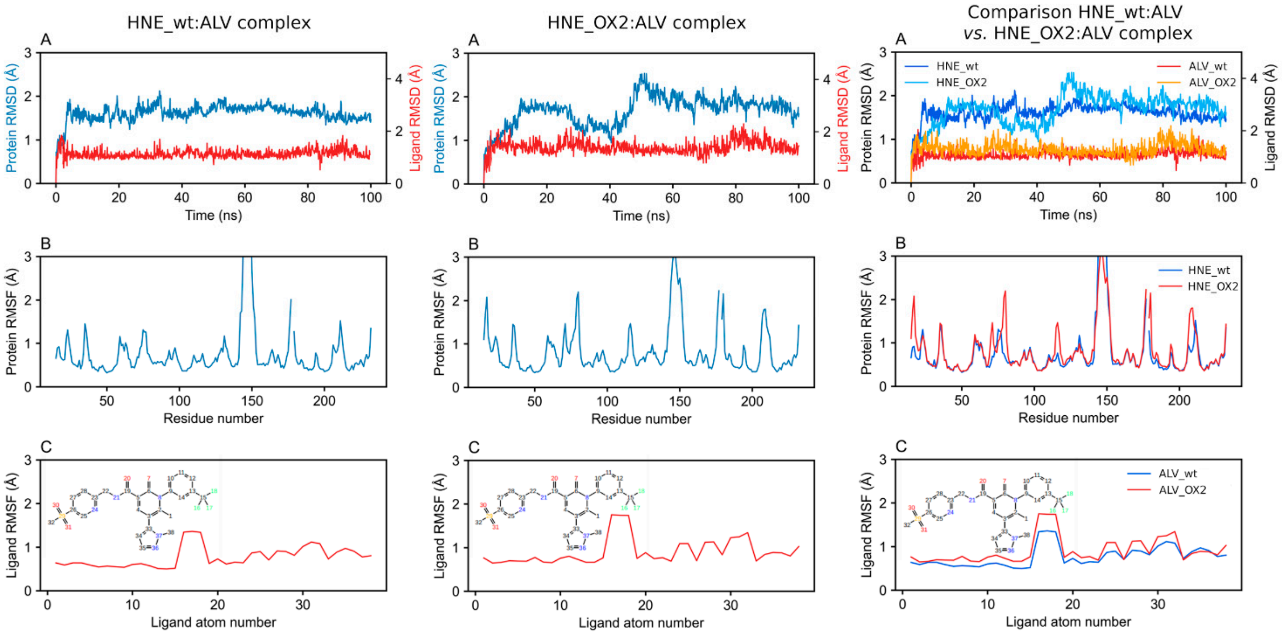

Figure S2. Molecular dynamics analysis of the HNE:Alvelestat (ALV) complex in wild-type (wt) and oxidized (HNE_OX2) forms. Panels A–C summarize structural stability and flexibility descriptors from 100 ns MD simulations. (A) Time evolution of protein Cα RMSD (blue) and ligand RMSD (red). (B) Residue-wise RMSF of HNE. (C) Ligand RMSF, reflecting internal mobility within the binding pocket. On the left column, the protein and ligand RMSD and RMSF for the HNE_wt:ALV complex are reported. On the center column, the protein and ligand RMSD and RMSF for the HNE_OX2:ALV complex are reported. On the right column, the comparison between the MD production for the native and oxidized models are superimposed. Overall, both systems show stable RMSD profiles, with the HNE_OX2 model displaying slightly increased local flexibility while preserving ligand binding stability.

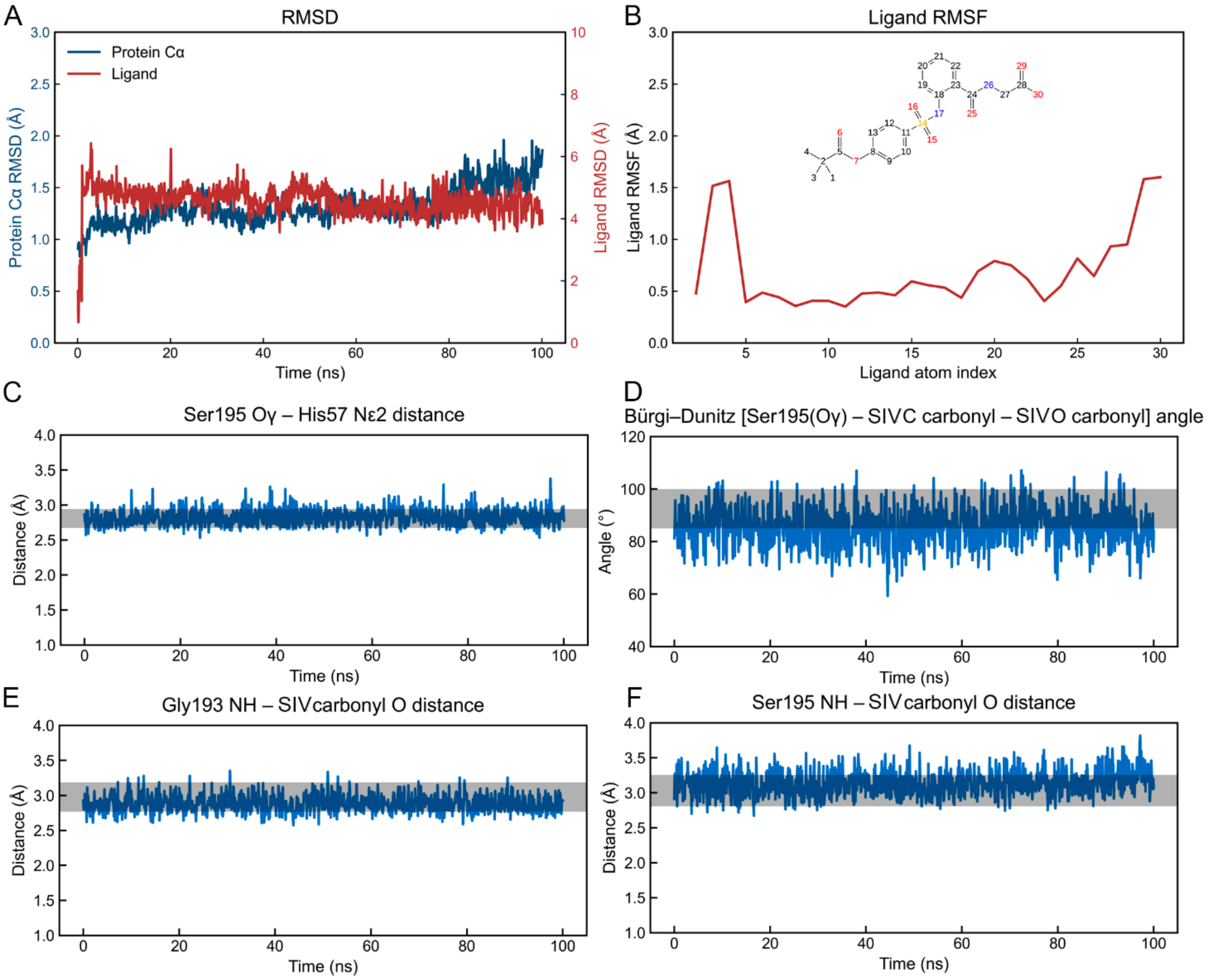

Figure S3. 100 ns MD simulation of the HNE_wt:SIV pre-complex. (A) Time evolution of protein Cα RMSD and ligand RMSD. (B) RMSF profile of sivelestat. (C–F) Time evolution of the Bürgi–Dunitz angle and of the distances between Ser195 Oγ–His57 Nε2, Gly193 NH–SIL carbonyl O, and Ser195 Oγ–SIL carbonyl O. Distances are reported in Å and the angle in degrees.

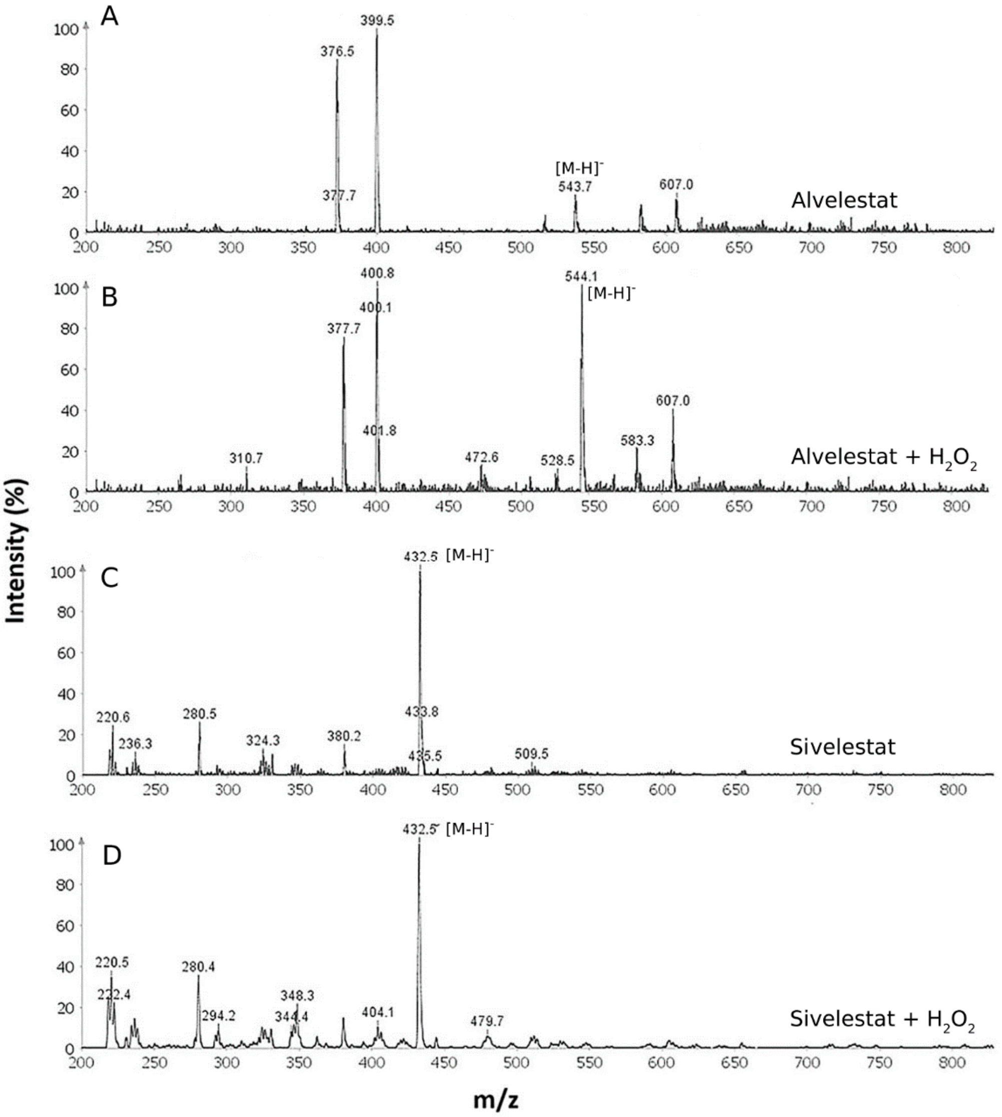

Figure S4. Mass spectra of Alvelestat and Sivelestat under control and oxidative conditions. Panels A–D correspond to Alvelestat, Alvelestat + H_2O_2, Sivelestat, and Sivelestat + H_2O_2, respectively.

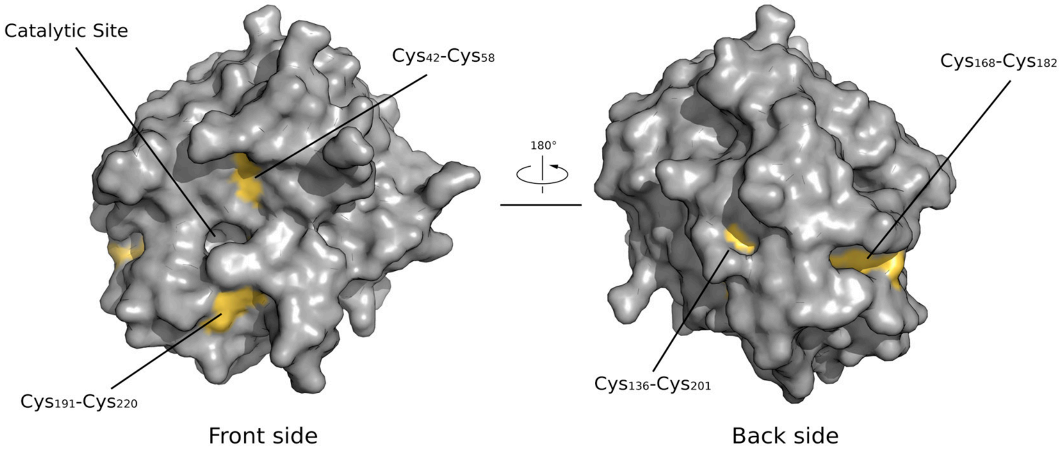

Figure S5. Surface representation of HNE highlighting the positions of the four disulfide bridges (yellow) relative to the catalytic site. Front (left) and back (right) views are shown, with Cys42–Cys58 and Cys191–Cys220 located near the catalytic cleft and S1 specificity pocket, and Cys136–Cys201 and Cys168–Cys182 positioned on the opposite face of the enzyme.

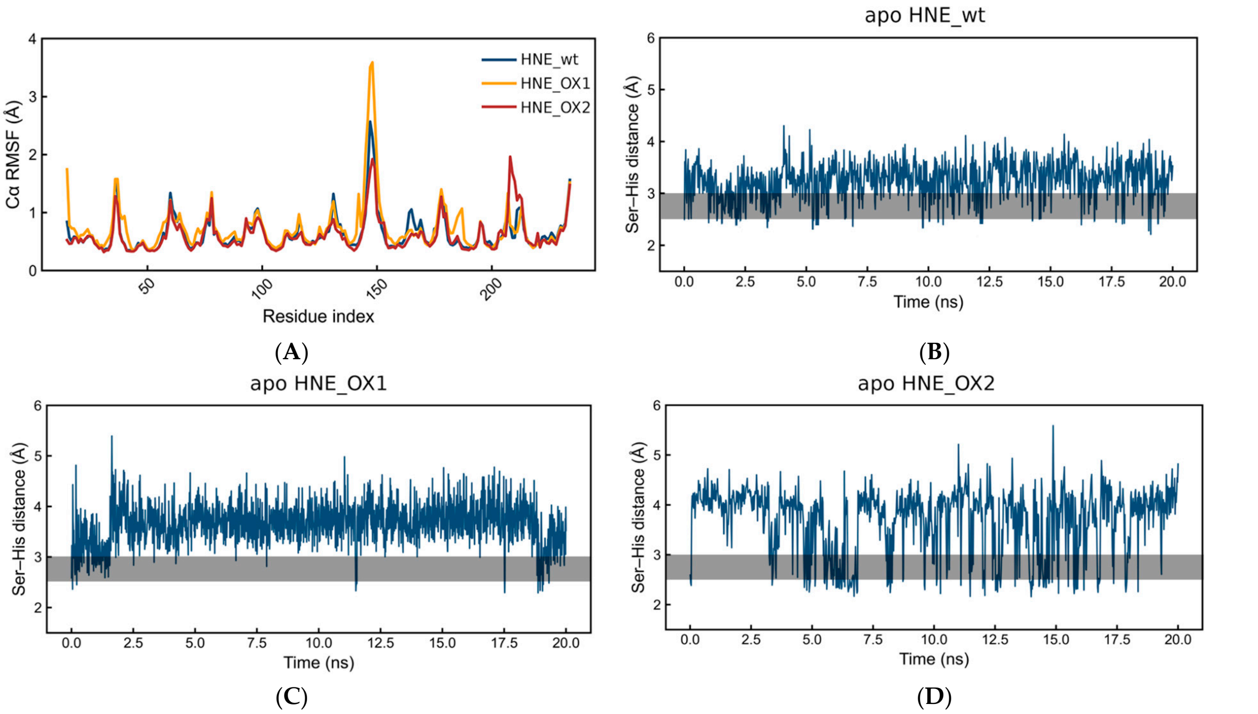

Figure S6. Effect of disulfide bond overoxidation on HNE flexibility and catalytic geometry. Superimposition of Cα RMSF profiles (A) and Ser195:Oγ–His57:Nε2 distance fluctuations over 20 ns MD simulations for HNE_wt (B) and oxidized models at the Cys42–Cys58 (HNE_OX1) (C) and Cys191–Cys220 (HNE_OX2) disulfide bridges (D). The productive range for Ser195:Oγ–His57:Nε2 distance is highlighted in gray.

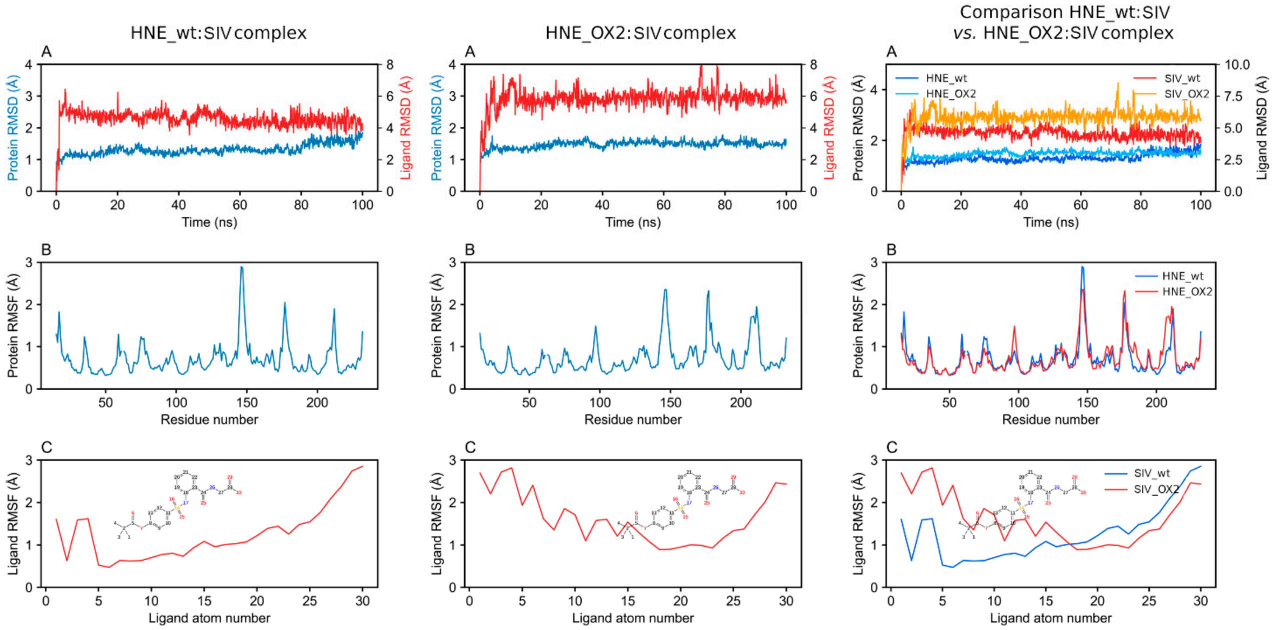

Figure S7. Molecular dynamics analysis of the HNE:Sivelestat (SIV) complex in wild-type (wt) and oxidized (HNE_OX2) forms. Panels A–C summarize structural stability and flexibility descriptors from 100 ns MD simulations. (A) Time evolution of protein Cα RMSD (blue) and ligand RMSD (red). (B) Residue-wise RMSF of HNE. (C) Ligand RMSF, reflecting internal mobility within the binding pocket. On the left column, the protein and ligand RMSD and RMSF for the HNE_wt:SIV complex are reported. On the center column, the protein and ligand RMSD and RMSF for the HNE_OX2:SIV complex are reported. On the right column, the comparison between the MD production for the native and oxidized models are superimposed. Overall, both systems show stable RMSD profiles, with the HNE_OX2 model displaying slightly increased local flexibility while preserving ligand binding stability. While the wt complex maintains a stable ligand

position, the HNE_OX2 model shows increased ligand mobility, particularly in the pivaloyl moiety (atoms 1–7), consistent with destabilization of the pre-acylation binding geometry upon oxidation of the Cys191–Cys220 disulfide bridge.
